# Oligosaccharins as Elicitors of Defense Responses in Wheat

**DOI:** 10.3390/polym13183105

**Published:** 2021-09-15

**Authors:** Laura Celina Ochoa-Meza, Eber Addí Quintana-Obregón, Irasema Vargas-Arispuro, Alejandro Bernardo Falcón-Rodríguez, Emmanuel Aispuro-Hernández, José J. Virgen-Ortiz, Miguel Ángel Martínez-Téllez

**Affiliations:** 1 Coordination of Food Technology of Vegetal Origin, Research Center for Food and Development (CIAD), Ave. Gustavo E. Astiazarán #46, Hermosillo 83304, Sonora, Mexico; laura.ochoa.dc19@estudiantes.ciad.mx (L.C.O.-M.); eaispuro@ciad.mx (E.A.-H.); 2 CONACYT—Research Center for Food and Development (CIAD), Hermosillo 83304, Sonora, Mexico; eber.quintana@ciad.mx (E.A.Q.-O.); jose.virgen@ciad.mx (J.J.V.-O.); 3 Coordination of Food Sciences, Research Center for Food and Development (CIAD), Hermosillo 83304, Sonora, Mexico; iris@ciad.mx; 4 Instituto Nacional de Ciencias Agrícolas (INCA), Carretera a Tapaste, km 3 1/2, San José de las Lajas 32700, Mayabeque, Cuba; alfalcon@inca.edu.cu; 5 Center of Innovation and Agroalimentary Development of Michoacán (CIDAM), Morelia 58341, Michoacán, Mexico

**Keywords:** wheat, biopolymers, chitosan oligomers, chitooligosaccharide, oligogalacturonide

## Abstract

Wheat is a highly relevant crop worldwide, and like other massive crops, it is susceptible to foliar diseases, which can cause devastating losses. The current strategies to counteract wheat diseases include global monitoring of pathogens, developing resistant genetic varieties, and agrochemical applications upon diseases’ appearance. However, the suitability of these strategies is far from permanent, so other alternatives based on the stimulation of the plants’ systemic responses are being explored. Plants’ defense mechanisms can be elicited in response to the perception of molecules mimicking the signals triggered upon the attack of phytopathogens, such as the release of plant and fungal cell wall-derived oligomers, including pectin and chitin derivatives, respectively. Among the most studied cell wall-derived bioelicitors, oligogalacturonides and oligochitosans have received considerable attention in recent years due to their ability to trigger defense responses and enhance the synthesis of antipathogenic compounds in plants. Particularly, in wheat, the application of bioelicitors induces lignification and accumulation of polyphenolic compounds and increases the gene expression of pathogenesis-related proteins, which together reduce the severity of fungal infections. Therefore, exploring the use of cell wall-derived elicitors, known as oligosaccharins, stands as an attractive option for the management of crop diseases by improving plant readiness for responding promptly to potential infections. This review explores the potential of plant- and fungal-derived oligosaccharins as a practical means to be implemented in wheat crops.

## 1. Introduction

The harvest of wheat in 2020 was around 757.6 million tons. For Central America and the Caribbean, the production was below the estimated average this year [1]. Wheat plays a dominant role in world food security since the Green Revolution [2]. However, the wheat crop suffers from various diseases, including fungal and bacterial infections, resulting in a significant drop in yield production and quality; additionally, wheat cultivation is significantly affected by climate change. Fungi of the genus *Puccinia* is a leading cause of yield loss in wheat globally: around 40% under favorable conditions. *Puccinia* fungus caused one of the most devastating outbreaks in Mexico, implicated in crop production losses up to 60% [3,4,5]. Fungal disease management typically relies on the chemical control and selection of resistant genotypes [6]. Synthetic fungicides, mainly triazoles like propiconazole, triadimefon, or fenpropimorph, are commonly used to treat cereal fungal diseases. This group of fungicides has low biodegradability and is consequently highly persistent in soil and water.

Furthermore, they disrupt endocrine function in mice, fish, and possibly humans [7,8]. Resistant genetic lines of wheat turn out to be a convenient option for the environment. However, the development cost is high, and the viability of their resistance is approximately three to five years, which renders them limited as a long-term solution [9]. Therefore, new approaches, preferably based on green methods, are needed to control fungal pathogens. It is worth considering an alternative to the standard control; as mentioned earlier, strategies use molecules that can emulate pathogens and induce the biosynthesis of antipathogenic compounds. Since these molecules elicit physiological, biochemical, and molecular responses, they are called elicitors.

Elicitors are capable of performing signaling functions in plants [10]. Biotic elicitors include microbial enzymes, fungal and bacterial lysates, yeast extracts, and polysaccharides from the cell walls of microorganisms (e.g., chitin and glucans), polysaccharides that arise from pathogen-drive degradation of the plant cell wall (pectin and its derivatives), intracellular proteins synthesized by the plant cell in response to different types of stresses or attack by pathogens, including plant hormones such as methyl jasmonate and salicylic acid (SA), their derivatives, and analogs [11].

Oligochitosans originate from the hydrolysis of chitosan, and oligogalacturonides are pectin-derived carbohydrate fragments resulting from hydrolysis of the middle lamella and primary cell wall of plants [12]. Some carbohydrate polymers, generally oligosaccharides or polysaccharides, have great potential as inducers of plant defense. The mechanism by which elicitors can protect plants is still far from fully understood, but considerable advances have been made. Plants possess a complex defense system that includes broad and specific responses such as accumulation of reactive oxygen species (ROS), increased expression of defense-related genes, activation of proteins that respond to pathogens, and the synthesis of phytoalexins and phytohormones. Such responses appear to be dependent on the plant species and type of elicitor. This review highlights wheat as one of the main vegetal models for its role in worldwide nourishment, and the potential use of oligosaccharin fragments derived from these carbohydrates either cell wall of plants or fungi have an elicitor activity in the systemic acquired resistance (SAR) in wheat as an alternative to decreasing the need for synthetic chemicals to protect this crop from diseases [13,14,15,16].

## 2. The Route of Plant Defense

The pathogen-associated molecular patterns (PAMP) are molecules with conserved features among pathogens that, upon perception, induce broad responses known to be the first line of defense of plants [17]. Plant pattern recognition receptors (PRRs) recognize PAMP and trigger a multifaceted immune response that increases tolerance to disease. In order to identify specific microbial epitopes on the cell surface, PRRs have to transit through intracellular compartments (from the endoplasmic reticulum through the Golgi apparatus to the trans-Golgi network, and finally to the plasma membrane). Therefore, the correct accumulation of PRRs in the plasma membrane is essential to trigger the immune response.

Furthermore, in the cell, PRRs are dynamically distributed through endocytic processes highly regulated [18]. Once PRRs are activated, they drive a series of biochemical and physiological responses to counteract pathogens called Pattern-Triggered Immunity (PTI), which involves mitogen-activated protein kinase signaling, certain modifications to histones, callose deposition, reactive oxygen species (ROS) explosion, and expression of pathogen-related genes [19].

PAMPs can be components of the cell wall, including chitin, β-glucans, ergosterol, and mannan, in the case of fungi. At the same time, bacteria-derived PAMPs include lipoic acid, peptidoglycans, and flagellin, which are highly conserved molecules essential for these microorganisms’ physiology and life cycle [20,21]. PAMP detection depends directly on PRR, which also possesses a group of conserved domains that include extracellular leucine-rich repeat receptor kinase or lysin motif domain and cytoplasmic kinase [17]. These domains are similar in structure to a family of plant transmembrane receptor-like kinases that are crucial for recognizing elicitor molecules, and thus for their effectiveness on the sequential expression of genes related to defense [22]; PAMPs triggers wall-associated kinase proteins, increasing the expression of genes involved in quantitative resistance (basal resistance) [23]. Upon PAMPs recognition, plants initiate a hypersensitive response (HR) process, a complex multicellular process commonly associated with programmed cell death in the presence of a pathogen. Although this HR mechanism leads to local cell death and tolerance to the pathogen, both processes are physiologically and genetically decoupled, e.g., the interaction between flax and *Melampsora lini* during flax rust developing [23].

If PRRs-mediated PAMPs perception fails, the pathogens depliage specialized effectors in the cell to suppress PTI by manipulating and modifying PTI components. Plants have developed a second layer of immunity, termed Effector Triggered Immunity (ETI), triggered when intracellular resistance proteins detect avirulent effectors in cooperation with accessory proteins. Indeed, there is a regulated process going from PTI to ETI. The ability of immune receptors to recognize appropriate ligands determines the magnitude of plant resistance, which is in turn associated with the development of an active immunity: when needed, PTI leads to a lengthy response, more robust activation of transcription factors, removal of harmful restrictions, and reinforcement of PTI pathways [24].

The phytohormones such as SA promote a potent response against pathogens that includes local reactions at the site of infection and systemic acquired resistance (SAR) activation. This mechanism protects plants from a broad spectrum of pathogens, including viruses, phytoplasma, bacteria, fungi, and nematodes [25]. Through SAR, the plant is self-defending against the colonization of additional pathogens by causing a systemic defense reaction that includes strengthening cell walls and producing pathogenesis-related proteins (PR) and phytoalexins [26].

ROS have been described as essential in the HR of plants, considering that oxidative burst is one of the first responses to pathogens attack [27]. An increase in NADPH oxidase enzymes produces ROS; furthermore, enzymes that detoxify ROS, such as catalase and ascorbate peroxidase, are suppressed by SA and nitric oxide to accumulate ROS and limit the pathogen’s advance beyond the site of infection. ROS participate in the activation of mitogen-activated protein kinase in plants. The redox state regulates NPR1 (Non-expressor of pathogenesis-related gen 1), an essential activator of SA-dependent defense responses such as SAR [28]. NPR1 accumulates in the cytosol as an inactive oligomer. Once reduced, it releases monomer units that migrate to the nucleus and interact with the reduced form of TGA1, a transcription factor belonging to the group of bZIP transcription factors that activate gene expression related to SA-dependent defense [29]. ROS can be regulatory molecules for establishing systemic defenses against pathogens [30].

There is a fragile balance in ROS concentration since their excess can be toxic to the cell and lead to cell death. Antioxidant enzymes such as guaiacol peroxidase, superoxide dismutase, enzymes of the glutathione ascorbate cycle, and catalase regulated ROS. Additionally, although not exclusively by ascorbate peroxidase, dehydroascorbate reductase, monodehydroascorbate reductase, and glutathione reductase. Other powerful non-enzymatic antioxidants in cells are ascorbate, glutathione, carotenoids, tocopherols, and phenolic compounds [31].

Plant infections, in addition to causing local responses (including ROS or HR), trigger SAR. Contrariwise, higher ROS levels also suppress SAR, and since nitric oxide and ROS operate in a feedback loop, ROS-mediated suppression of SAR may involve nitric oxide, corroborating that an excessive accumulation of ROS could negatively regulate SAR [32]. During SAR, the signaling function shows similarities between SA- and nitric oxide-triggered networks. Hence, crosstalk between SA- and nitric oxide-dependent pathways might provide multiple points to coregulate these pathways. Thereby, facilitating a tighter regulation could explain the differential gene expression induced in response to varying nitric oxide donor concentrations and SAR suppression seen in plants with high nitric oxide levels [33].

During SAR, the plant alarm signals are spread by the phloem, mainly containing two hormones, SA and jasmonic acid (JA), to transmit the alert to distal parts of the infection sites, allowing plants to deal more quickly and to counteract biotic and abiotic stresses [33,34] efficiently. In such enhanced cells, only in the presence of a pathogen, defense compounds are synthesized. SA and JA maintain a mutually antagonistic relationship [34], although some cases where they have synergistic interactions have been reported [35]. The crosstalk between jasmonate and SA of *Arabidopsis* is notorious. Other phytohormones such as ethylene and abscisic acid regulate JA inactivation by SA or vice versa [36].

Typically, SA activity is associated with biotrophic pathogens, as in aphid attacks, the activation of SAR in plants like *Arabidopsis* and wheat. A family of transcription factors called Non-Expressor Genes Related to Pathogenesis (NPR) directly controls the transcription of PR-genes associated with the SA signaling pathway. In particular, NPR1 is considered the master regulator of SA signaling [37,38]. It is stipulated that SAR depends on SA. SAR is induced in wheat and tobacco plants either by an inducer or a pathogen. The foliage of tobacco plants requires the production of phenylpropanoid pathway compounds for the efficient development of SAR. Also, de novo production of phenylpropanoids can be induced by SAR, which in wheat promotes a higher content of antioxidants in the bran [39].

## 3. Salicylic Acid: The Hormone of Plant Defense

Among endogenous plant growth regulators, salicylic acid is a unique phenolic class compound with an essential role in modulating various physiological and metabolic processes, such as growth regulation, thermogenesis, ethylene biosynthesis, flower induction, seed germination and ion absorption by roots, stomatal movements, inhibition of leaf abscission, accumulation of chlorophyll and carotenoids, photosynthesis, enzyme activation, and plant maturation [40,41]. SA-mediated immune responses involve strengthening the cell wall by increasing lignin and callose production, synthesizing antimicrobial secondary metabolites like phytoalexins, and synthesizing antimicrobial proteins like glucanases and chitinases, which may degrade the cell walls of pathogens [42]. 

SA has a role as a regulator of pathogens recognition. In plants, the endogenous content of SA and exogenous application of SA can trigger immune-like responses. SA levels control the transcriptional reprogramming via the perception of NPR proteins with SA protein regulators; thus, positive and negative transcriptional regulation of SA biosynthesis is required for fine-tuning. SA levels for optimal defense without causing unnecessary fitness costs [43]. Salicylic acid signaling contributes to the tolerance to *Fusarium graminearum* in wheat and barley due to the constitutive expression of NPR1 and NPR1-like genes [44].

SA biosynthesis occurs inside the chloroplasts, which can be carried out mainly by two different metabolic pathways. One is derived from L-phenylalanine and catalyzed by the enzyme phenylalanine ammonium lyase (PAL). Simultaneously, the second one, the enzyme isochorismate synthase (ICS), uses chorismate as a precursor. How isochorismate is converted to SA is still unclear, and several are missing links between immune receptors and the activation of SA biosynthesis [45]. Both pathways start with chorismic acid. The end product of the shikimic acid pathway, an alternative route to *ICS*, intervenes in SA production. In some dicotyledonous plant models, such as *Arabidopsis*, *Nicotiana benthamiana*, and tomato, most synthesized SA occurs via chorismate [46,47]. In wheat, the predominant mechanisms by which SA is biosynthesized are still to be determined. However, under freezing stress, wheat, cucumber, and watermelon synthesize SA through PAL, but it is uncertain if, under biotrophic stress, wheat synthesizes SA through PAL or ICS [48]; meanwhile, in other angiosperms, such as strawberry and soja under biotrophic stress, SA biosynthesis occurs through both routes; ISC and PAL. Moreover, in strawberries, *chorismate mutase*, *salicylate hydroxylase*, and *isochorismate synthase* act as effector genes related to the homeostasis of SA during host/pathogen interaction between strawberry and *C. fructicola.*

The plant ICSs show quite distinct biochemical properties despite their sequence similarities. These differences between ICSs biochemical properties in plants may reflect the plant species-related particularities in SA production [49], as well as the complex homeostasis of SA, in which both pathways may act cooperatively [50,51].

## 4. Types of Elicitors

Plants have the adaptability of priming; this prepares them to respond faster and more vigorously to the stress caused by the attack of pathogens. Elicitors generate the priming state, availed to activate plant responses such as the production of phytohormones and phytoalexins, an increase in the expression of defense-related genes, and enhanced synthesis of enzymes with antioxidant activity [52]. According to its nature, the elicitor can be considered a biotic or abiotic driving factor. Abiotics include metal salts, physical factors, or UV light. At the same time, biotics is far more diverse due to the immense nature of chemical molecules produced or derived from biological agents with eliciting activities. 

Table 1 shows different biotic elicitors applied to wheat or various crops in the last decade to counteract stresses caused by pathogens or climatic and edaphological conditions. The activity and effectivity of these elicitors have been evaluated in diverse developmental stages of plants, such as seed, vegetative growth, fruit development, and postharvest storage, using different application methods.

### 4.1. Eliciting Phytohormones

The phytohormones are small organic molecules that can influence physiological processes in plants at low concentrations. They participate in several functions during the different stages of plant development and senescence and help plants cope with abiotic and biotic stresses throughout their life cycle [71]. Some phytohormones fulfill a double function as a resource against pathogens and also as signaling agents. Salicylic acid is one of them since its synthesis increases upon fungal attack [72]. The application of JA and SA to wheat leaves promoted the activity of β-1,3-glucanases and thaumatin-like proteins that significantly reduced (up to 56%) the disease incited by *Stagonospora nodorum*. Hence, the increased resistance in wheat to *S. nodorum* after SA and JA application may be related to the accumulation of PR-proteins [73].

Salicylic acid influences a series of physiological processes such as plant growth. It improved the level of the antioxidant system (catalase, peroxidase, superoxide dismutase, and proline) when applied to mung beans exposed to aluminum stress. In such a study, the increase in antioxidants was accompanied by decreased H_2_O_2_ content and peroxidation of membrane lipids, suggesting that SA detoxifies oxidative stress induced by Aluminum. Improved plant growth reflects stress resistance, photosynthetic pigments, photosynthesis-related attributes, and membrane stability index [41]. Wheat treated with 100 mM SA accumulated higher levels of osmolytes and transcripts related to stress-associated genes. In addition, SA treatment increased the total antioxidant capacity of wheat. It reduced the detrimental effect of heat shock on soluble starch synthase activity as the synthesis of starch granules, which may have relevant implications for adapting crops to heat [74]. In recent years, SA and other phytohormones have been used alone or combined with different biotic elicitors as inductors of biochemical and physiological responses associated with the upregulation in essential defense-related expression genes wheat (Table 2).

### 4.2. Eliciting Phytochemicals and Plant-Derived Compounds

Various compounds that come from plants can act as elicitors, among which we can find volatile compounds, carbohydrates, saponins, proteins, peptides, and lipids. The food industry uses λ-carrageenan from red algae as an additive that could also protect tomato plants by triggering JA-related gene expression [84].

Volatile organic compounds from plants can work as elicitors since many of these compounds are produced and released by attacking herbivores or pathogens. In maize seedlings, the green leaf volatiles as Z-3-hexenyl acetate (Z-3-HAC) can create a priming agent against herbivorous insects.

Other phytochemicals that can be used as elicitors are saponins. The (25*R*)-5α-spirostane-2α,3β,5α-triol 3-*O*-(*O*-α-L-rhamnopyranosyl-(1→2)-*O*-(β-d-galactopyra-nosyl-(1→3))-β-d-glucopyranoside) saponin isolated from *Agapanthus africanus* elicits defense responses of plants. It exerts direct antifungal activity and stimulates the in vitro peroxidase enzyme activity in wheat [56].

Furthermore, the plant-derived cytokinins can promote *Arabidopsis* resistance to *Pseudomonas syringae* pv. *tomato* DC3000 since the cytokinin activated transcription factor ARR2 contributes specifically to the tolerance to *P. syringae* pv. *tomato*. The SA response factor TGA3 binds to ARR2, and cytokinin modulates SA signaling to reduce susceptibility to *P. syringae* pv. *tomato*. Therefore, applying exogenous cytokinins to plants could be a strategy to increase their tolerance to pathogens [85]. 

Cell walls are mainly composed of carbohydrates, proteins, and aromatic compounds. Pectins are among these carbohydrates comprising a complex family of polysaccharides, including homogalacturonan, xylogalacturonan, and apiogalacturonan [12]. The partial degradation of homogalacturonan releases oligogalacturonides (OGAS), oligomers of alpha-1,4-linked galacturonosyl residues [86,87]. OGAS can elicit defense responses, including the accumulation of ROS and PR proteins, and protect plants against pathogen infection. In addition, OGAS is recognized by Wall Assoc. Kinase (WAK) receptor in *N. tabacum* and *Vitis vinifera* [24].

### 4.3. Microorganism-Derived Elicitors

Some molecules derived from microorganism’s cells show activity as elicitors, e.g., a protein named PeFOC1 isolated from *Fusarium oxysporum*. This protein triggers the immune response and systemic acquired resistance in tobacco by inducing early reaction events and HR in tobacco cells. In addition, PeFOC1 regulated PR gene expression, activated SA and JA/ethylene signaling pathways, and caused the deposition of callose and phenolic compounds in tobacco as evidence of SAR induction [62]. Another example of the elicitor capacity of microorganisms-derived compounds is PeBL2, a protein isolated from *Brevibacillus laterosporus* A60, which triggered an early defensive response in *N. benthamiana* revealed by ROS accumulation (H_2_O_2_ and O_2_^−^). In the systemic resistance of tobacco against *Botrytis cinerea*, PeBL2 plays a vital role in the induction of defense-related early events, as well as an HR [61]. The cutinase from *Sclerotinia sclerotiorum* also triggers defense responses in tobacco plants by inducing an HR in leaves and producing many signaling molecules and secondary metabolites involved in plant resistance, including the PR proteins PR1a, PR2b, and lipoxygenase [88].

Chitin is a linear long-chain homo-polymer composed of N-acetyl glucosamine units [poly(N-acetyl-β-D-glucosamine)] with acetamide groups at the C-2 positions in place of the hydroxyl groups in the repeating unit of the macromolecular chain [89]. Although commercial chitin is obtained mainly from crustaceans’ exoskeletons, fungi cell walls are also a rich source of this compound. The fungus–plant warfare releases chitin fragments. This mechanism is evolutionarily maintained, making chitin one of the most outstanding PAMPs. In tobacco, the Lysin domain of plant cells recognizes chitin fragments by an extracellular kinase (CHRK1), eliciting the plant defense response and inducing the biosynthesis of ROS, phytoalexins, and protein phosphorylation [90]. The cell surface receptors of chitin, CERK1 (chitin elicitor receptor kinase 1), LYK5 (lysin motif receptor kinase 5), and CEBiP (chitin elicitor binding protein) recognize chitin and its derivatives (chitosan and chitooligosaccharides) in *A. thaliana* and rice, while the receptors LYR4 and LYK9 exert this chitin recognizing function in soybean, and LYM2 and CERK in other legumes [24,91,92,93]. Despite the vast availability of chitin, its extreme insolubility is a significant problem confronting the development of processes and uses based on this compound [89]. 

The first derivative of chitin is chitosan, a linear polysaccharide consisting of 2-acetamido-2-deoxy-β-d-glucose (N-acetylglucosamine) and 2-amino-2-deoxy-β-d-glucose (N-glucosamine) units linked by β-glycosidic bonds (1–4), which can have different proportions of N-acetylglucosamine [94]. The presence of amino groups in the molecule structure converts this polymer into a natural cationic polyelectrolyte with pKa~6.5, which gives it particular properties [95]. Amongst polysaccharides and natural polymers, the cationic nature of chitosan is peculiar, and most applications can be related to this feature. Chitosan has unique characteristics such as biocompatibility and biodegradability and possesses reactive functional groups that make it useful in different areas. It shows a series of bioactive properties: analgesic, antimicrobial (antibacterial, antifungal), antioxidant, anti-inflammatory, antacid, hypolipidemic, antidiabetic, anticancer, antitumor, and bioadhesive [89]. 

It can effectively maintain the quality of fruits and vegetables and control postharvest decomposition during storage and shelf life. Chitosan is a known inductor of plant defense reactions. It can also be used in multi-component edible coatings, providing the desired protection barrier for the fruit, and serving as a vehicle to incorporate specific additives such as minerals, vitamins, essential oils, and other nutraceutical compounds [96]. Chitosan activates the defense processes in plant cells, chelation of metals, and suppresses the supply and assimilation of essential nutrients for microbial growth. Likewise, the positive charge of the amino groups of chitosan could increase its degree of deacetylation, which accrues the antimicrobial properties of chitosan [96].

Chitosan has also been used in vegetable crops such as potato, in which foliar applications of high molecular weight chitosan (200–558 mg/ha) and hydrolyzed chitosan achieved 15% to 30% better yield [97]. In peach, chitosan, and oligochitosans, treatments delayed fruit softening and senescence. They showed to effectively control brown rot due to an increase in the production of the antioxidant enzyme catalase, chitinase, glucanase, and the expression of the *peroxidase* and *glucanase* genes [98].

An exciting feature of chitosan is the excellent biological activity exerted by its fractions with a lower molecular weight called chitooligosaccharides (COS) [99]. The poor solubility of unmodified chitosan in organic solvents also limits its utilization [100]. Unlike chitosan, its hydrolyzed products (chitosan oligomers or COS) quickly dissolve in water due to their short-chain lengths and free amino groups found in the D-glucosamine unit [101]. COS exerts bioactive properties such as antiviral, antibacterial, and antioxidant activities. They are growth promoters and decrease the susceptibility of plants to abiotic stress damage [102]. COS concentration and degree of acetylation are essential in determining their inducing activity. The biological activity of chitin fragments is dependent on the degree of polymerization, with the highest reported activity for the degree of polymerization = 7 or 8 and little or no activity for small oligomers, with a degree of polymerization < 5 [103].

COS favors the production of secondary metabolites in plants. In addition, they induce ion flow, ROS production, activation of mitogen-activated protein kinases, expression of defense genes, synthesis of phytoalexins, strengthening of the cell wall, and in some cases, induction of cell death [104].

The application of COS solution in rice seedlings decreased the severity of *Pyricularia grisea* and increased the enzymatic activity of PAL, glucanase, and chitinase [105]. In soybean, both chitosan and chitin oligosaccharides increased the enzymatic activity of PAL and tyrosine ammonium lyase, enzymes that actively participate in the defense mechanisms of plants [104]. Likewise, the application of COS increased resistance to *C. gloeosporioides* in navel oranges by increasing the activity of PAL enzymes, chitinase, peroxidase, and the accumulation of glycoproteins rich in hydroxyproline, improving orange quality during storage as related to a decreased susceptibility to anthracnose [106].

## 5. Elicitors and Their Effect on Wheat

Wheat is a crop of economic relevance and a model to approach technological alternatives in agriculture. The use of elicitors to enhance wheat production and quality through activation of defense responses against phytopathogens is not an exception; therefore, a wide range of elicitor types have been studied (Table 2). 

Inorganic substances such as ozone induce wheat responses, increasing antioxidant enzyme activities; lipid peroxidation; H_2_O_2_ and Ca^+2^ levels; also decreasing *Blumeria graminis* f. sp. *tritici* invasion [79]. Sodium bicarbonate is another inorganic substance that has been evaluated in wheat to activate plant defense against *Fusarium culmorum* (Table 1) [78]. 

Non-pathogenic endophytic bacteria promote the protection of plants against the attack of pathogens. Inoculation of wheat seeds of the “Alixan” and “Altigo” varieties with *Paenibacillus* sp. strain B2 induced systemic resistance in wheat and suppressed the level of infection by *M. graminicola* more than 59%. The genes involved in wheat basal defense pathways, ROS production, and the synthesis of flavonoids, SA, and JA, appear to play a vital role in the resistance to *M. graminicola* [107]. Wheat seedlings treated with a liquid microbial fermentation derivative (MFP) product obtained from a mixture of bacteria and yeast showed reduced powdery mildew pustules and increased PR genes’ expression. It is essential to develop alternative methods to conventional fungicides to counteract resistance development. For instance, agar supplemented with MFP inhibited the germination and differentiation of powdery mildew. Therefore, an MFP elicitor may provide an effective method to control fungi development in wheat [76].

The infiltration of SA in wheat leaves modified lipid metabolism by stimulating the synthesis of phosphatidic acid and inducing the octadecanoid pathway, changing the set of fatty acids, and reducing the content of unsaturated fatty acids caused partial resistance against *Blumeria graminis* f. sp. *tritici* [75]. The combination of phytohormones and other elicitors was functional in the acquisition of freezing tolerance in winter wheat. GXAG (carbohydrate elicitor composed of monosaccharides of the cell wall) has demonstrated a synergistic effect when applied before ABA, which suggests the ability of GXAG to increase cell receptivity to ABA signaling.

Durum wheat seeds were treated with chitosan and inoculated with *Fusarium graminearum* fungus that causes root rot. An increased concentration of total phenols and enhanced enzymatic activity of PAL, polyphenol oxidase, guaiacol peroxidase, and ascorbate peroxidase reduced the root rot disease severity in greenhouse and field conditions. Chitosan did not affect the germination performance of wheat. On the contrary, the application of chitosan is an alternative against soil pathogens in wheat seeds and seedlings [108]. 

The exogenous application of COS in wheat seeds improved shoot and root lengths in both fresh and dry weight of wheat seedlings. The decreased lipid peroxidation of the membrane also increased the chlorophyll content and caused an increase in the antioxidant activity of superoxide dismutase, peroxidase, catalase, and ascorbate peroxidase. Interestingly COS affected the tolerance of wheat seedlings to cold stress strictly related to their DP [102]. In addition, the application of COS to wheat seedlings increased the enzymatic activity of chitinase and glucanase. COS with a polymerization degree from three to four units exerted a more potent effect at higher dose concentrations [109]. Moreover, COS is recognized as PAMPs by potential PRRs: W5G2U8, W5HY42, and W5I0R4 proteins that function as COS binding sites in wheat, suggesting possible interactions between plasma membrane proteins on the surface of the plasma membrane of the wheat protoplast [110].

OGAS decreased the level of *Blumeria graminis* f. sp. *tritici* infection in wheat as a decrease in haustoria formation and an increase in the accumulation of autofluorescent compounds in papillae but did not activate PAL [111].

## 6. Future Perspective and Limitations

The current trend in product development is to generate regenerative products, meaning that they do not contribute to the collection or extraction of raw materials from nature. COS can be obtained from chitin extracted from the exoskeletons of shrimp, which have become a problematic residue in shrimp-producing zones. Meanwhile, OGAS can be obtained with a good yield from pectin obtained from citric residues. Therefore, the production of both elicitors as active ingredients supposes an alternative use for food industry by-products worldwide and contributes to the circular economy model. Initially, the low performance of obtaining COS was a significant limitation for large-scale applications. However, the processes and techniques for obtaining good quality oligosaccharins at high yields, either by chemical or enzymatic methods, are becoming efficient over time.

Nowadays, there are patented products on the market, such as fytosave^®^, whose active substance is a complex of COS-OGAS. According to the low-risk phytosanitary category, these products are included in Europe’s list of active substances. The fytosave^®^ shows effectivity in potatoes and rice [54,59,60]. To date, no application of this product in grains has been reported.

The biotic nature of oligosaccharins allows them to be used in organic farming. However, a possible limitation on the use of these products is that both OGAS and COS are potential carbon sources for microorganisms. In wheat, it is still unknown if upregulation of defense-associated gene expression by the effect of oligosaccharins is able to induce resistance and trigger the production of phytoalexins, increase the concentration of plant defense-associated phytohormones, and if the application of these elicitors is an efficient strategy to the overall stimulation of systemic acquired resistance.

## 7. Conclusions

The application of plant- and fungal-derived oligosaccharides has improved resistance to various pathogens in plants such as potato, rice, orange, and tomato, which highlights the oligosaccharins potential to develop practical means to be implemented in the production of wheat crops. The elicitation mechanisms of oligosaccharins in plants include the accumulation of ROS and glycoproteins rich in hydroxyproline and the induction of the expression of genes coding for the PR proteins PI-1, PR-1, acid PR-2, PAL, peroxidase, and for proteins required for the synthesis of SA. Wheat has specific receptors of COS and OGAS that allow their recognition as PAMP molecules and then trigger signaling similar to that which occurs when attacked by a biotrophic pathogen, thus activating the defense system in an environmentally friendly manner for sustainable plant protection.

## Figures and Tables

**Table 1:** Examples of chemical/biological elicitors in crops.

Crop	Stressor	Elicitor	Elicitor Type	Concentration	Mode of Application	Triggered Response	Reference
Tomato	*Ralstonia solanacearum*	SA	Phytohormone	1 µM	Soaked seeds	Increased the activities of peroxidase and polyphenol oxidase enzymes	[53]
Tomato	*Leveillula taurica*	COS + OGA	Fragments of cell wall + fungal cell wall	50 ppm	Foliar spray	Upregulation of PR proteins and salicylic acid (SA)-related genes	[54]
Rice	*Xanthomonas oryzae* pv. *Oryzae*	Methyl salicylate	Phytohormone	75 and 100 mg L^−1^	Soaked seeds	Promoted early growth and provided better protection against diseases	[55]
Wheat	*Fusarium oxysporum.*	Saponin isolated from *Agapanthus africanus*	Phytochemical	125 µg mL^−1^	Foliar spray	Stimulation of peroxidase enzyme activity	[56]
Citrus	Low temperature	Pectic oligosaccharides	Fragments of cell wall	10 g L^−1^	Postharvest spray application on fruits	Early defense signals	[57]
Wheat	Low temperature	GXAG + ABA	Fragments of cell wall	5 µg mL^−1^ (GXAG) + 50 µM (ABA)	Application in roots	Initiation of freezing tolerance acquisition in winter plants	[58]
Potato	*Phytophthora infestans*	FytoSave (COS-OGAS)	Fungal cell wall—Fragments of cell wall	12.5 g L^−1^	Foliar spray	Upregulation of defense genes *PI-1*, *PR-1*, and *acidic PR-2* in potato	[59]
Rice	*Meloidogyne graminicola*					Induced defense dependent on *OsPAL4* gene expression in rice	[60]
Tobacco	*Botrytis cinerea*	PeBL2	Microorganism-Derived	50 µM	Infiltrated leaves	Generation of ROS (H_2_O_2_ and O_2_^−^) and systemic resistance activation	[61]
Tobacco	tobacco mosaic virus and *Pseudomonas syringae* pv. *tabaci*.	PeFOC1	Microorganism-Derived	5 µM	Infiltrated leaves	Upregulation of *NtPAL*, *NtEDS1*, *NtLOX*, and *NtPDF*, activated SA and JA/Et signaling pathways, induced callose, and phenolic compounds deposition	[62]
Avocado	*Colletotrichum gloeosporioides*	Chitosan	Fungal cell wall	16 mg mL^−1^	In vitro	Induce specific accumulation of phenylpropanoids and an antifungal diene	[63]
Tomato	*Cucumber mosaic virus* (CMV)	Chitosan	Fungal cell wall	10 mL. plant^−1^	Foliar spray	Reduced viral load and upregulated *PAL5* expression.	[64]
Citrus	*Geotrichum candidum*	SA	Phytohormone	2.5 mmol L^−1^	Wounded fruit	Enhanced phenylpropanoid pathway-related enzyme activities and stimulated the synthesis of phenolic acids and lignin	[65]
		*P. membranaefaciens*	Microorganism-derivate	1 × 10^8^ cells mL^−1^			
		Chitooligosaccharide	Fungal cell wall	15 g L^−1^			
Arabidopsis	*Pseudomonas syringae* pv. *tomato*	Cellobiose	Fragments of cell wall	100 µM	In vitro	Signaling similar to other PAMPs/DAMPs,	[66]
Pea	*Aphanomyces euteiches*	Oligogalacturonides	Fragments of cell wall	80 µg	Injected plants	Stimulated defense mechanisms, including the SA pathway	[67]
Rice	*Sogatella furcifera*	4-Fluorophenoxyacetic	Synthetic chemical	0.5 to 5 mg. L^−1^	Root application and Foliar spray	Modulated the production of peroxidases, H_2_O_2,_ and flavonoids	[68]
Apricot	Low temperature	SA + COS	Phytohormones+ fungal cell wall	SA (1 mmol L^−1)^ + 0.05% COS (*w/v*)	Foliar spray	Reduced chilling injury and improved fruit quality	[69]
Grapefruits	*Penicillium digitatum*	SA + Chitosan	Phytohormones+ fungal cell wall	SA (2 mM) + 10 g L^−1^ chitosan (*w/v*)	Fruit dipped	Enhanced the chitinase, β-1,3-glucanase, peroxidase, phenylalanine ammonia-lyase, and polyphenoloxidase activities and stimulated the synthesis of total phenolic compounds content	[70]

**Table 2:** The effect of elicitors on the regulation of defense-related genes expression in wheat.

Pathogen	Elicitor	Mode of Application	Gene Up-Regulation	Suggested Mechanisms	Reference
*Blumeria graminis* f. sp. *tritici*	SA	Foliar infiltration	PI-PLC2, LOX,	Induction of the octadecanoid pathway	[75]
*Stagonospora nodorum*.	SA/JA	Foliar spray	GLU, TLPs	Induced in response to infection	[73]
*Blumeria graminis* f. sp. *tritici*	MFP	Foliar spray	PR1, PR4, PR5, and PR9	Induction of plant defense systems	[76]
*Sitobion avenae*	PeaT1	Seed immersion and foliar spray	ICS, PR1,	Increased number of trichomes and higher accumulation of wax. Induced SA and JA levels	[77]
*Fusarium culmorum*	Sodium bicarbonate	Seed immersion	B2H2, PAL	Induction of plant defense systems	[78]
*Blumeria graminis* f. sp. *tritici*	Ozone	Gas	PR1, LOX, PAL	Expression induced via the SA pathway	[79]
*Blumeria graminis* f. sp. *tritici*	Saccharin/PBZ	Foliar spray	PR1.1, PR2, PR4, CHI3 CHI4, TaNPR1, PAL, LOX, AOS, WCI2, WCI3, WRKY72a/b e	Induced expression of defense-related genes, including a WRKY-type transcription factor. Increased SA and JA biosynthesis	[80]
*Fusarium graminearum*	SA	Soil drench	PAL	Activated antioxidant defense responses and possible induced systemic acquired resistance	[81]
*Zymoseptoria tritici*	λ-Carrageenan	Foliar spray	PR1, PR4, PR5, PR8,13-lipoxygenase 2, PAL, PR15	Displayed antimicrobial activities, increased antioxidative processes, and plant defense signaling of SA and JA	[82]
	Cytosine-phosphate guanine oligodesoxynucleotide motifs (CpG ODN)	Foliar spray	PR4, PR5,13-lipoxygenase 2		
	*Spirulina platensis*	Foliar spray	PR1,13-lipoxygenase 2, PAL, PR15		
	Glycine betaine	Foliar spray	PR4, PR5,13-lipoxygenase 2, PAL, PR15		
	Ergosterol	Foliar spray	PR1, PR4, PR5, PR8,13-lipoxygenase 2, PAL, PR15		
*Fusarium graminearum*	Green Leaf Volatile Z-3-Hexenyl Acetate	Cuvette System	PR1, PR4, PR5, peroxidase	Enhanced defense against the hemibiotrophic fungus *F. graminearum*, resulting in slower disease progress, reduced symptom development, and lower fungal growth	[83]

## Data Availability

No data were used to support this study.
